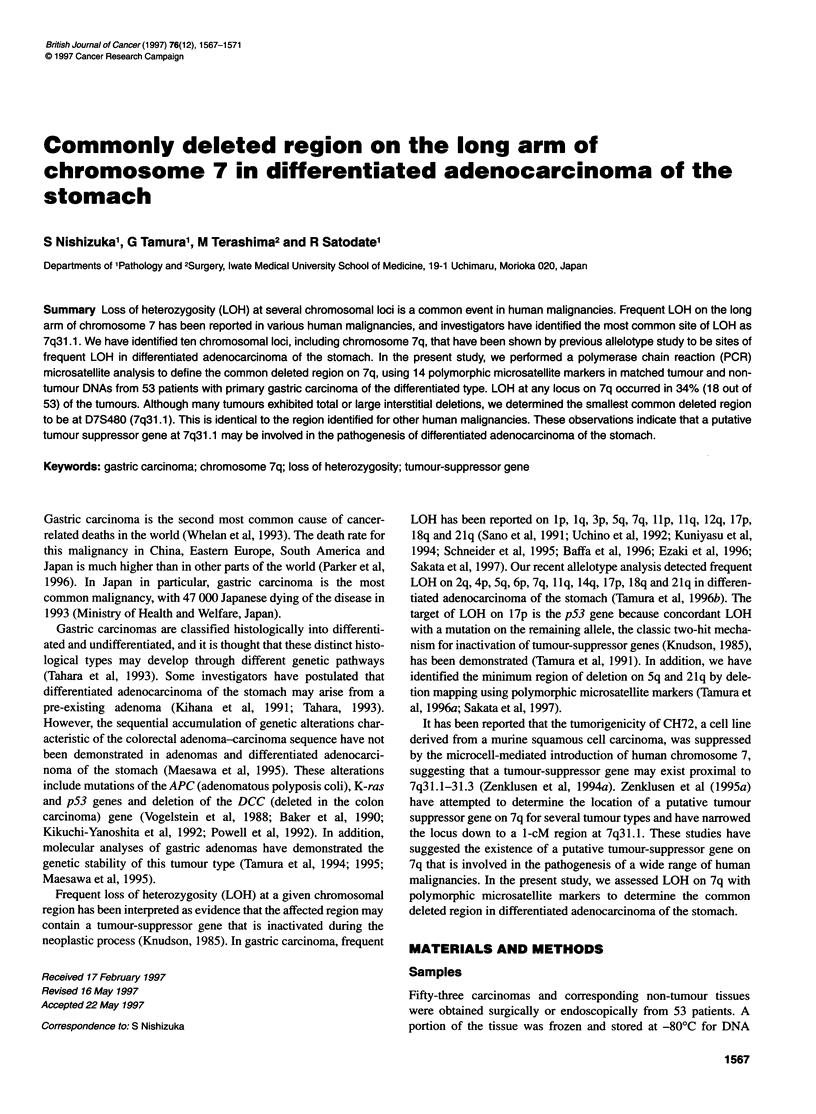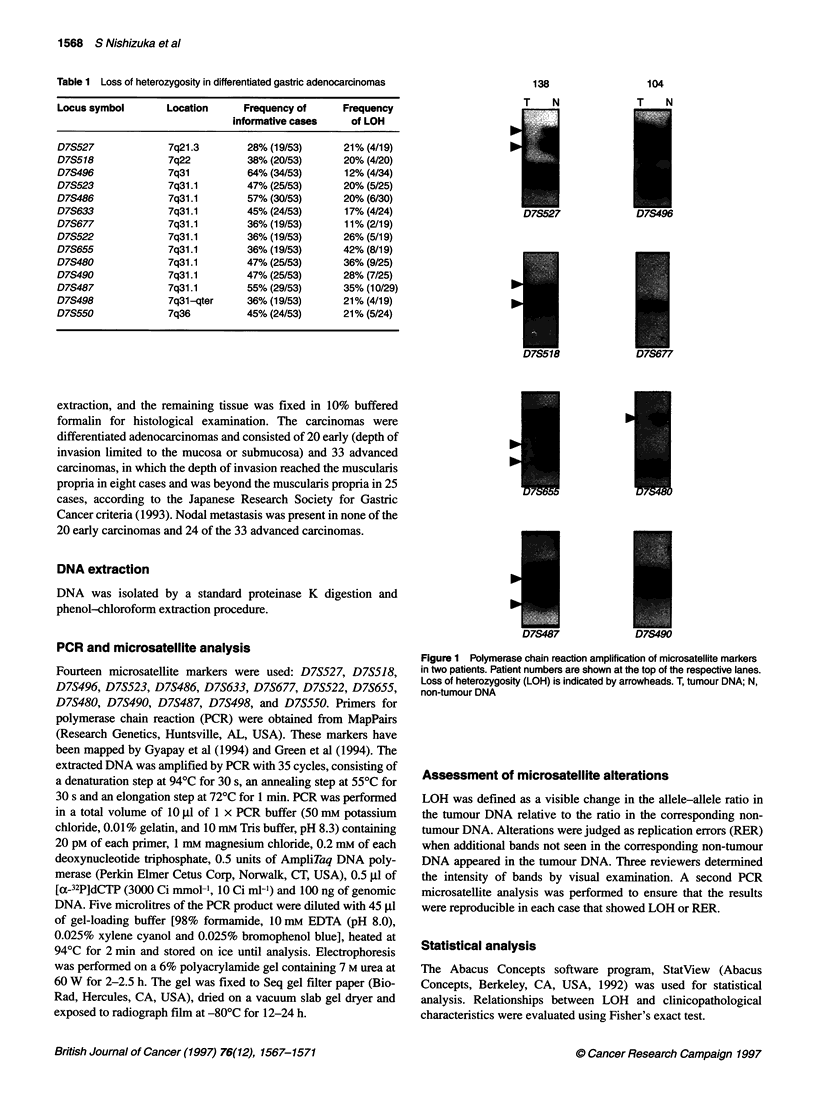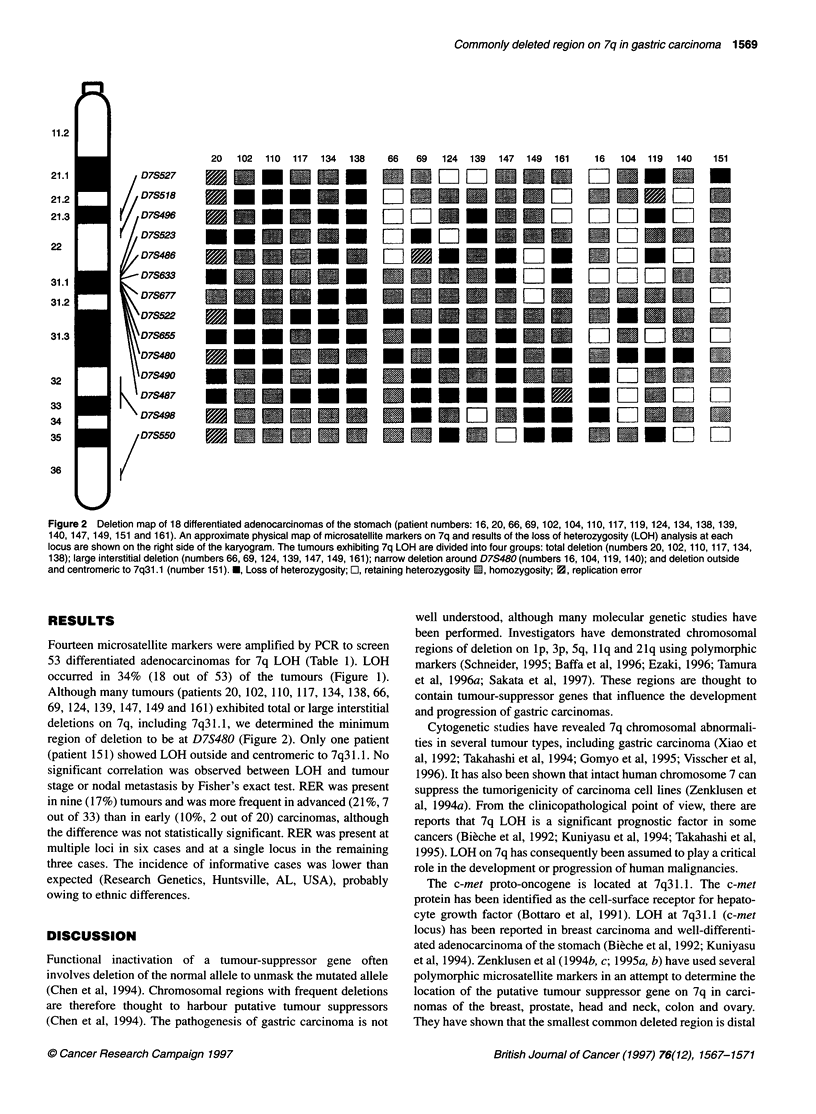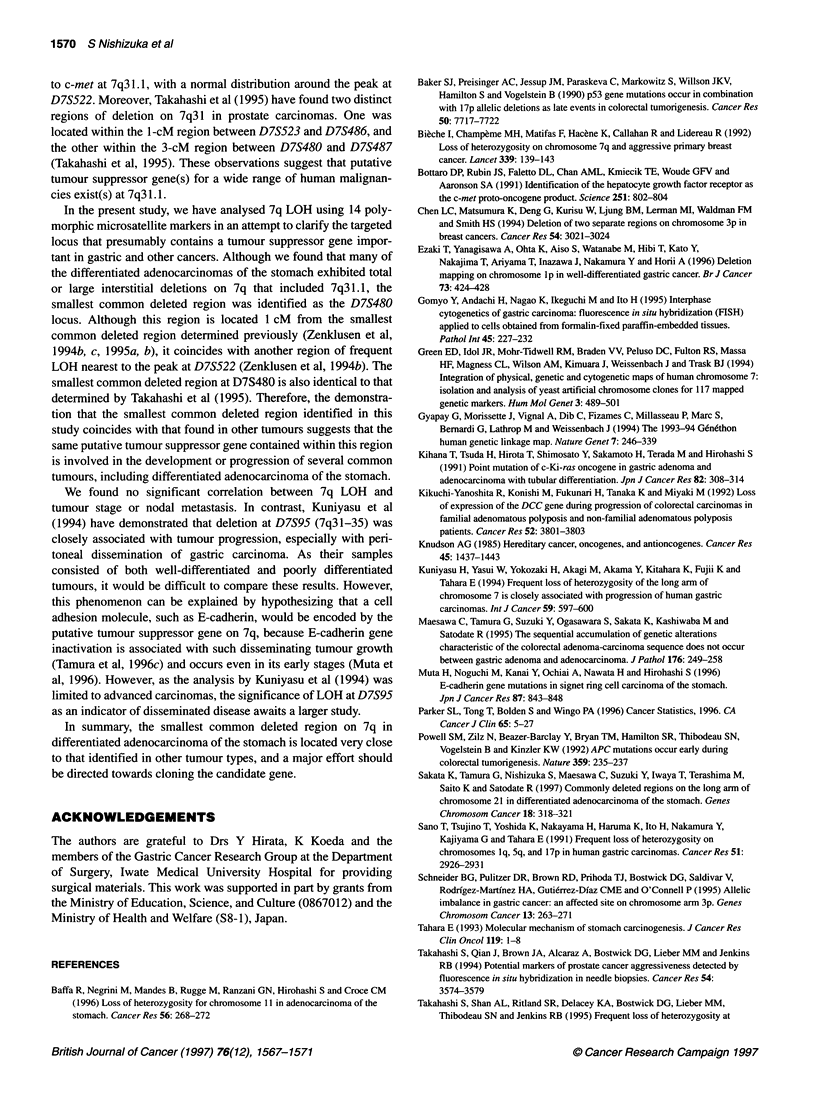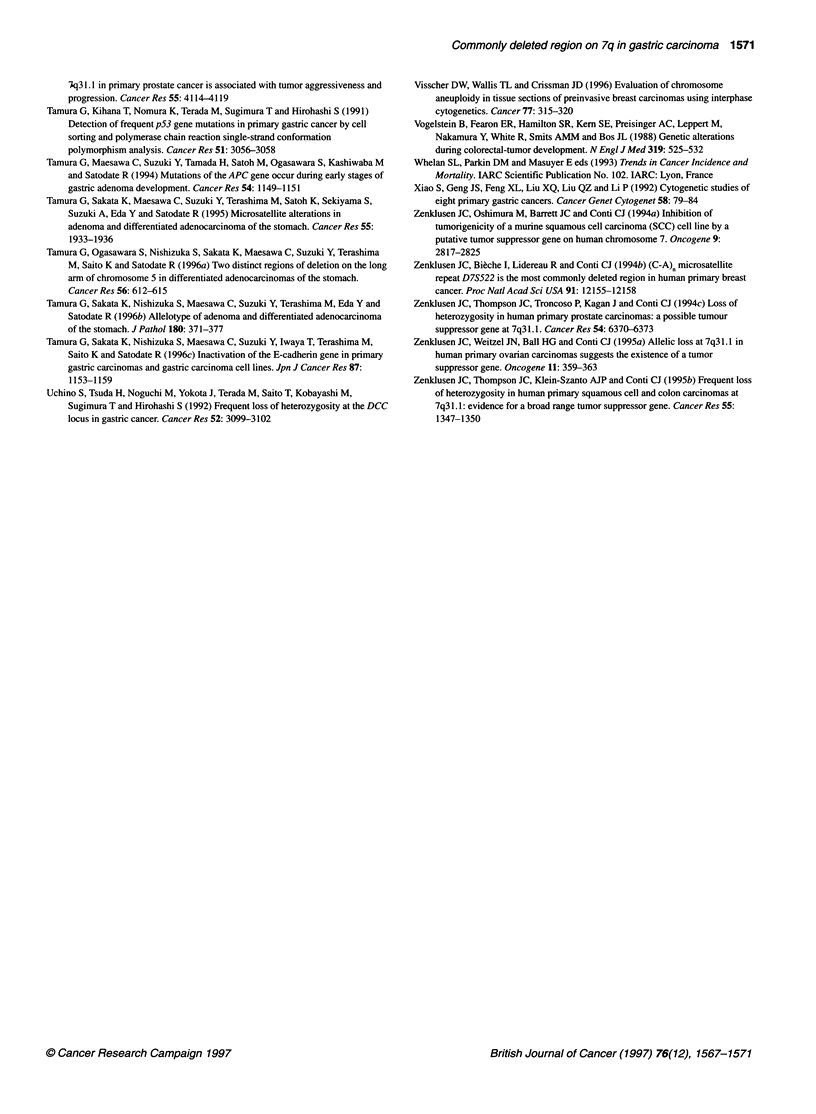# Commonly deleted region on the long arm of chromosome 7 in differentiated adenocarcinoma of the stomach.

**DOI:** 10.1038/bjc.1997.598

**Published:** 1997

**Authors:** S. Nishizuka, G. Tamura, M. Terashima, R. Satodate

**Affiliations:** Department of Pathology, Iwate Medical University School of Medicine, Morioka, Japan.

## Abstract

**Images:**


British Journal of Cancer (1997) 76(12), 1567–1571
© 1997 Cancer Research Campaign

### Commonly deleted region on the long arm of chromosome 7 in differentiated adenocarcinoma of the stomach

**S Nishizuka[1], G Tamura[1], M Terashima[2] and R Satodate[1]**

Departments of [1]Pathology and [2]Surgery, Iwate Medical University School of Medicine, 19-1 Uchimaru, Morioka 020, Japan

**Summary** Loss of heterozygosity (LOH) at several chromosomal loci is a common event in human malignancies. Frequent LOH on the long arm of chromosome 7 has been reported in various human malignancies, and investigators have identified the most common site of LOH as 7q31.1. We have identified ten chromosomal loci, including chromosome 7q, that have been shown by previous allelotype study to be sites of frequent LOH in differentiated adenocarcinoma of the stomach. In the present study, we performed a polymerase chain reaction (PCR) microsatellite analysis to define the common deleted region on 7q, using 14 polymorphic microsatellite markers in matched tumour and non-tumour DNAs from 53 patients with primary gastric carcinoma of the differentiated type. LOH at any locus on 7q occurred in 34% (18 out of 53) of the tumours. Although many tumours exhibited total or large interstitial deletions, we determined the smallest common deleted region to be at D7S480 (7q31.1). This is identical to the region identified for other human malignancies. These observations indicate that a putative tumour suppressor gene at 7q31.1 may be involved in the pathogenesis of differentiated adenocarcinoma of the stomach.

**Keywords:** gastric carcinoma; chromosome 7q; loss of heterozygosity; tumour-suppressor gene

Gastric carcinoma is the second most common cause of cancer-related deaths in the world (Whelan et al, 1993). The death rate for this malignancy in China, Eastern Europe, South America and Japan is much higher than in other parts of the world (Parker et al, 1996). In Japan in particular, gastric carcinoma is the most common malignancy, with 47 000 Japanese dying of the disease in 1993 (Ministry of Health and Welfare, Japan).

Gastric carcinomas are classified histologically into differentiated and undifferentiated, and it is thought that these distinct histological types may develop through different genetic pathways (Tahara et al, 1993). Some investigators have postulated that differentiated adenocarcinoma of the stomach may arise from a pre-existing adenoma (Kihana et al, 1991; Tahara, 1993). However, the sequential accumulation of genetic alterations characteristic of the colorectal adenoma–carcinoma sequence have not been demonstrated in adenomas and differentiated adenocarcinoma of the stomach (Maesawa et al, 1995). These alterations include mutations of the *APC* (adenomatous polyposis coli), K-*ras* and *p53* genes and deletion of the *DCC* (deleted in the colon carcinoma) gene (Vogelstein et al, 1988; Baker et al, 1990; Kikuchi-Yanoshita et al, 1992; Powell et al, 1992). In addition, molecular analyses of gastric adenomas have demonstrated the genetic stability of this tumour type (Tamura et al, 1994; 1995; Maesawa et al, 1995).

Frequent loss of heterozygosity (LOH) at a given chromosomal region has been interpreted as evidence that the affected region may contain a tumour-suppressor gene that is inactivated during the neoplastic process (Knudson, 1985). In gastric carcinoma, frequent LOH has been reported on 1p, 1q, 3p, 5q, 7q, 11p, 11q, 12q, 17p, 18q and 21q (Sano et al, 1991; Uchino et al, 1992; Kuniyasu et al, 1994; Schneider et al, 1995; Baffa et al, 1996; Ezaki et al, 1996; Sakata et al, 1997). Our recent allelotype analysis detected frequent LOH on 2q, 4p, 5q, 6p, 7q, 11q, 14q, 17p, 18q and 21q in differentiated adenocarcinoma of the stomach (Tamura et al, 1996*b*). The target of LOH on 17p is the *p53* gene because concordant LOH with a mutation on the remaining allele, the classic two-hit mechanism for inactivation of tumour-suppressor genes (Knudson, 1985), has been demonstrated (Tamura et al, 1991). In addition, we have identified the minimum region of deletion on 5q and 21q by deletion mapping using polymorphic microsatellite markers (Tamura et al, 1996*a*; Sakata et al, 1997).

It has been reported that the tumorigenicity of CH72, a cell line derived from a murine squamous cell carcinoma, was suppressed by the microcell-mediated introduction of human chromosome 7, suggesting that a tumour-suppressor gene may exist proximal to 7q31.1–31.3 (Zenklusen et al, 1994*a*). Zenklusen et al (1995*a*) have attempted to determine the location of a putative tumour suppressor gene on 7q for several tumour types and have narrowed the locus down to a 1-cM region at 7q31.1. These studies have suggested the existence of a putative tumour-suppressor gene on 7q that is involved in the pathogenesis of a wide range of human malignancies. In the present study, we assessed LOH on 7q with polymorphic microsatellite markers to determine the common deleted region in differentiated adenocarcinoma of the stomach.

### MATERIALS AND METHODS

### Samples

Fifty-three carcinomas and corresponding non-tumour tissues were obtained surgically or endoscopically from 53 patients. A portion of the tissue was frozen and stored at –80°C for DNA

*Received 17 February 1997*
*Revised 16 May 1997*
*Accepted 22 May 1997*

*Correspondence to:* S Nishizuka

**Table 1** Loss of heterozygosity in differentiated gastric adenocarcinomas

| Locus symbol | Location | Frequency of informative cases | Frequency of LOH |
|---|---|---|---|
| *D7S527* | 7q21.3 | 28% (19/53) | 21% (4/19) |
| *D7S518* | 7q22 | 38% (20/53) | 20% (4/20) |
| *D7S496* | 7q31 | 64% (34/53) | 12% (4/34) |
| *D7S523* | 7q31.1 | 47% (25/53) | 20% (5/25) |
| *D7S486* | 7q31.1 | 57% (30/53) | 20% (6/30) |
| *D7S633* | 7q31.1 | 45% (24/53) | 17% (4/24) |
| *D7S677* | 7q31.1 | 36% (19/53) | 11% (2/19) |
| *D7S522* | 7q31.1 | 36% (19/53) | 26% (5/19) |
| *D7S655* | 7q31.1 | 36% (19/53) | 42% (8/19) |
| *D7S480* | 7q31.1 | 47% (25/53) | 36% (9/25) |
| *D7S490* | 7q31.1 | 47% (25/53) | 28% (7/25) |
| *D7S487* | 7q31.1 | 55% (29/53) | 35% (10/29) |
| *D7S498* | 7q31–qter | 36% (19/53) | 21% (4/19) |
| *D7S550* | 7q36 | 45% (24/53) | 21% (5/24) |

extraction, and the remaining tissue was fixed in 10% buffered formalin for histological examination. The carcinomas were differentiated adenocarcinomas and consisted of 20 early (depth of invasion limited to the mucosa or submucosa) and 33 advanced carcinomas, in which the depth of invasion reached the muscularis propria in eight cases and was beyond the muscularis propria in 25 cases, according to the Japanese Research Society for Gastric Cancer criteria (1993). Nodal metastasis was present in none of the 20 early carcinomas and 24 of the 33 advanced carcinomas.

### DNA extraction

DNA was isolated by a standard proteinase K digestion and phenol–chloroform extraction procedure.

### PCR and microsatellite analysis

Fourteen microsatellite markers were used: *D7S527*, *D7S518*, *D7S496*, *D7S523*, *D7S486*, *D7S633*, *D7S677*, *D7S522*, *D7S655*, *D7S480*, *D7S490*, *D7S487*, *D7S498*, and *D7S550*. Primers for polymerase chain reaction (PCR) were obtained from MapPairs (Research Genetics, Huntsville, AL, USA). These markers have been mapped by Gyapay et al (1994) and Green et al (1994). The extracted DNA was amplified by PCR with 35 cycles, consisting of a denaturation step at 94°C for 30 s, an annealing step at 55°C for 30 s and an elongation step at 72°C for 1 min. PCR was performed in a total volume of 10 μl of 1 × PCR buffer (50 mM potassium chloride, 0.01% gelatin, and 10 mM Tris buffer, pH 8.3) containing 20 pM of each primer, 1 mM magnesium chloride, 0.2 mM of each deoxynucleotide triphosphate, 0.5 units of Ampli*Taq* DNA polymerase (Perkin Elmer Cetus Corp, Norwalk, CT, USA), 0.5 μl of [α-$^{32}$P]dCTP (3000 Ci mmol$^{-1}$, 10 Ci ml$^{-1}$) and 100 ng of genomic DNA. Five microlitres of the PCR product were diluted with 45 μl of gel-loading buffer [98% formamide, 10 mM EDTA (pH 8.0), 0.025% xylene cyanol and 0.025% bromophenol blue], heated at 94°C for 2 min and stored on ice until analysis. Electrophoresis was performed on a 6% polyacrylamide gel containing 7 M urea at 60 W for 2–2.5 h. The gel was fixed to Seq gel filter paper (Bio-Rad, Hercules, CA, USA), dried on a vacuum slab gel dryer and exposed to radiograph film at –80°C for 12–24 h.

**Figure 1** Polymerase chain reaction amplification of microsatellite markers in two patients. Patient numbers are shown at the top of the respective lanes. Loss of heterozygosity (LOH) is indicated by arrowheads. T, tumour DNA; N, non-tumour DNA

### Assessment of microsatellite alterations

LOH was defined as a visible change in the allele–allele ratio in the tumour DNA relative to the ratio in the corresponding non-tumour DNA. Alterations were judged as replication errors (RER) when additional bands not seen in the corresponding non-tumour DNA appeared in the tumour DNA. Three reviewers determined the intensity of bands by visual examination. A second PCR microsatellite analysis was performed to ensure that the results were reproducible in each case that showed LOH or RER.

### Statistical analysis

The Abacus Concepts software program, StatView (Abacus Concepts, Berkeley, CA, USA, 1992) was used for statistical analysis. Relationships between LOH and clinicopathological characteristics were evaluated using Fisher's exact test.

© Cancer Research Campaign 1997

**Figure 2** Deletion map of 18 differentiated adenocarcinomas of the stomach (patient numbers: 16, 20, 66, 69, 102, 104, 110, 117, 119, 124, 134, 138, 139, 140, 147, 149, 151 and 161). An approximate physical map of microsatellite markers on 7q and results of the loss of heterozygosity (LOH) analysis at each locus are shown on the right side of the karyogram. The tumours exhibiting 7q LOH are divided into four groups: total deletion (numbers 20, 102, 110, 117, 134, 138); large interstitial deletion (numbers 66, 69, 124, 139, 147, 149, 161); narrow deletion around *D7S480* (numbers 16, 104, 119, 140); and deletion outside and centromeric to 7q31.1 (number 151). ■, Loss of heterozygosity; □, retaining heterozygosity ▩, homozygosity; ▨, replication error

### RESULTS

Fourteen microsatellite markers were amplified by PCR to screen 53 differentiated adenocarcinomas for 7q LOH (Table 1). LOH occurred in 34% (18 out of 53) of the tumours (Figure 1). Although many tumours (patients 20, 102, 110, 117, 134, 138, 66, 69, 124, 139, 147, 149 and 161) exhibited total or large interstitial deletions on 7q, including 7q31.1, we determined the minimum region of deletion to be at *D7S480* (Figure 2). Only one patient (patient 151) showed LOH outside and centromeric to 7q31.1. No significant correlation was observed between LOH and tumour stage or nodal metastasis by Fisher's exact test. RER was present in nine (17%) tumours and was more frequent in advanced (21%, 7 out of 33) than in early (10%, 2 out of 20) carcinomas, although the difference was not statistically significant. RER was present at multiple loci in six cases and at a single locus in the remaining three cases. The incidence of informative cases was lower than expected (Research Genetics, Huntsville, AL, USA), probably owing to ethnic differences.

### DISCUSSION

Functional inactivation of a tumour-suppressor gene often involves deletion of the normal allele to unmask the mutated allele (Chen et al, 1994). Chromosomal regions with frequent deletions are therefore thought to harbour putative tumour suppressors (Chen et al, 1994). The pathogenesis of gastric carcinoma is not well understood, although many molecular genetic studies have been performed. Investigators have demonstrated chromosomal regions of deletion on 1p, 3p, 5q, 11q and 21q using polymorphic markers (Schneider, 1995; Baffa et al, 1996; Ezaki, 1996; Tamura et al, 1996*a*; Sakata et al, 1997). These regions are thought to contain tumour-suppressor genes that influence the development and progression of gastric carcinomas.

Cytogenetic studies have revealed 7q chromosomal abnormalities in several tumour types, including gastric carcinoma (Xiao et al, 1992; Takahashi et al, 1994; Gomyo et al, 1995; Visscher et al, 1996). It has also been shown that intact human chromosome 7 can suppress the tumorigenicity of carcinoma cell lines (Zenklusen et al, 1994*a*). From the clinicopathological point of view, there are reports that 7q LOH is a significant prognostic factor in some cancers (Bièche et al, 1992; Kuniyasu et al, 1994; Takahashi et al, 1995). LOH on 7q has consequently been assumed to play a critical role in the development or progression of human malignancies.

The c-*met* proto-oncogene is located at 7q31.1. The c-*met* protein has been identified as the cell-surface receptor for hepatocyte growth factor (Bottaro et al, 1991). LOH at 7q31.1 (c-*met* locus) has been reported in breast carcinoma and well-differentiated adenocarcinoma of the stomach (Bièche et al, 1992; Kuniyasu et al, 1994). Zenklusen et al (1994*b*, *c*; 1995*a*, *b*) have used several polymorphic microsatellite markers in an attempt to determine the location of the putative tumour suppressor gene on 7q in carcinomas of the breast, prostate, head and neck, colon and ovary. They have shown that the smallest common deleted region is distal

© Cancer Research Campaign 1997

to c-*met* at 7q31.1, with a normal distribution around the peak at *D7S522*. Moreover, Takahashi et al (1995) have found two distinct regions of deletion on 7q31 in prostate carcinomas. One was located within the 1-cM region between *D7S523* and *D7S486*, and the other within the 3-cM region between *D7S480* and *D7S487* (Takahashi et al, 1995). These observations suggest that putative tumour suppressor gene(s) for a wide range of human malignancies exist(s) at 7q31.1.

In the present study, we have analysed 7q LOH using 14 polymorphic microsatellite markers in an attempt to clarify the targeted locus that presumably contains a tumour suppressor gene important in gastric and other cancers. Although we found that many of the differentiated adenocarcinomas of the stomach exhibited total or large interstitial deletions on 7q that included 7q31.1, the smallest common deleted region was identified as the *D7S480* locus. Although this region is located 1 cM from the smallest common deleted region determined previously (Zenklusen et al, 1994*b*, *c*, 1995*a*, *b*), it coincides with another region of frequent LOH nearest to the peak at *D7S522* (Zenklusen et al, 1994*b*). The smallest common deleted region at D7S480 is also identical to that determined by Takahashi et al (1995). Therefore, the demonstration that the smallest common deleted region identified in this study coincides with that found in other tumours suggests that the same putative tumour suppressor gene contained within this region is involved in the development or progression of several common tumours, including differentiated adenocarcinoma of the stomach.

We found no significant correlation between 7q LOH and tumour stage or nodal metastasis. In contrast, Kuniyasu et al (1994) have demonstrated that deletion at *D7S95* (7q31–35) was closely associated with tumour progression, especially with peritoneal dissemination of gastric carcinoma. As their samples consisted of both well-differentiated and poorly differentiated tumours, it would be difficult to compare these results. However, this phenomenon can be explained by hypothesizing that a cell adhesion molecule, such as E-cadherin, would be encoded by the putative tumour suppressor gene on 7q, because E-cadherin gene inactivation is associated with such disseminating tumour growth (Tamura et al, 1996*c*) and occurs even in its early stages (Muta et al, 1996). However, as the analysis by Kuniyasu et al (1994) was limited to advanced carcinomas, the significance of LOH at *D7S95* as an indicator of disseminated disease awaits a larger study.

In summary, the smallest common deleted region on 7q in differentiated adenocarcinoma of the stomach is located very close to that identified in other tumour types, and a major effort should be directed towards cloning the candidate gene.

### ACKNOWLEDGEMENTS

The authors are grateful to Drs Y Hirata, K Koeda and the members of the Gastric Cancer Research Group at the Department of Surgery, Iwate Medical University Hospital for providing surgical materials. This work was supported in part by grants from the Ministry of Education, Science, and Culture (0867012) and the Ministry of Health and Welfare (S8-1), Japan.

### REFERENCES

Baffa R, Negrini M, Mandes B, Rugge M, Ranzani GN, Hirohashi S and Croce CM (1996) Loss of heterozygosity for chromosome 11 in adenocarcinoma of the stomach. *Cancer Res* **56**: 268–272

Baker SJ, Preisinger AC, Jessup JM, Paraskeva C, Markowitz S, Willson JKV, Hamilton S and Vogelstein B (1990) p53 gene mutations occur in combination with 17p allelic deletions as late events in colorectal tumorigenesis. *Cancer Res* **50**: 7717–7722

Bièche I, Champème MH, Matifas F, Hacène K, Callahan R and Lidereau R (1992) Loss of heterozygosity on chromosome 7q and aggressive primary breast cancer. *Lancet* **339**: 139–143

Bottaro DP, Rubin JS, Faletto DL, Chan AML, Kmiecik TE, Woude GFV and Aaronson SA (1991) Identification of the hepatocyte growth factor receptor as the c-*met* proto-oncogene product. *Science* **251**: 802–804

Chen LC, Matsumura K, Deng G, Kurisu W, Ljung BM, Lerman MI, Waldman FM and Smith HS (1994) Deletion of two separate regions on chromosome 3p in breast cancers. *Cancer Res* **54**: 3021–3024

Ezaki T, Yanagisawa A, Ohta K, Aiso S, Watanabe M, Hibi T, Kato Y, Nakajima T, Ariyama T, Inazawa J, Nakamura Y and Horii A (1996) Deletion mapping on chromosome 1p in well-differentiated gastric cancer. *Br J Cancer* **73**: 424–428

Gomyo Y, Andachi H, Nagao K, Ikeguchi M and Ito H (1995) Interphase cytogenetics of gastric carcinoma: fluorescence *in situ* hybridization (FISH) applied to cells obtained from formalin-fixed paraffin-embedded tissues. *Pathol Int* **45**: 227–232

Green ED, Idol JR, Mohr-Tidwell RM, Braden VV, Peluso DC, Fulton RS, Massa HF, Magness CL, Wilson AM, Kimuara J, Weissenbach J and Trask BJ (1994) Integration of physical, genetic and cytogenetic maps of human chromosome 7: isolation and analysis of yeast artificial chromosome clones for 117 mapped genetic markers. *Hum Mol Genet* **3**: 489–501

Gyapay G, Morissette J, Vignal A, Dib C, Fizames C, Millasseau P, Marc S, Bernardi G, Lathrop M and Weissenbach J (1994) The 1993–94 Généthon human genetic linkage map. *Nature Genet* **7**: 246–339

Kihana T, Tsuda H, Hirota T, Shimosato Y, Sakamoto H, Terada M and Hirohashi S (1991) Point mutation of c-Ki-*ras* oncogene in gastric adenoma and adenocarcinoma with tubular differentiation. *Jpn J Cancer Res* **82**: 308–314

Kikuchi-Yanoshita R, Konishi M, Fukunari H, Tanaka K and Miyaki M (1992) Loss of expression of the *DCC* gene during progression of colorectal carcinomas in familial adenomatous polyposis and non-familial adenomatous polyposis patients. *Cancer Res* **52**: 3801–3803

Knudson AG (1985) Hereditary cancer, oncogenes, and antioncogenes. *Cancer Res* **45**: 1437–1443

Kuniyasu H, Yasui W, Yokozaki H, Akagi M, Akama Y, Kitahara K, Fujii K and Tahara E (1994) Frequent loss of heterozygosity of the long arm of chromosome 7 is closely associated with progression of human gastric carcinomas. *Int J Cancer* **59**: 597–600

Maesawa C, Tamura G, Suzuki Y, Ogasawara S, Sakata K, Kashiwaba M and Satodate R (1995) The sequential accumulation of genetic alterations characteristic of the colorectal adenoma-carcinoma sequence does not occur between gastric adenoma and adenocarcinoma. *J Pathol* **176**: 249–258

Muta H, Noguchi M, Kanai Y, Ochiai A, Nawata H and Hirohashi S (1996) E-cadherin gene mutations in signet ring cell carcinoma of the stomach. *Jpn J Cancer Res* **87**: 843–848

Parker SL, Tong T, Bolden S and Wingo PA (1996) Cancer Statistics, 1996. *CA Cancer J Clin* **65**: 5–27

Powell SM, Zilz N, Beazer-Barclay Y, Bryan TM, Hamilton SR, Thibodeau SN, Vogelstein B and Kinzler KW (1992) *APC* mutations occur early during colorectal tumorigenesis. *Nature* **359**: 235–237

Sakata K, Tamura G, Nishizuka S, Maesawa C, Suzuki Y, Iwaya T, Terashima M, Saito K and Satodate R (1997) Commonly deleted regions on the long arm of chromosome 21 in differentiated adenocarcinoma of the stomach. *Genes Chromosom Cancer* **18**: 318–321

Sano T, Tsujino T, Yoshida K, Nakayama H, Haruma K, Ito H, Nakamura Y, Kajiyama G and Tahara E (1991) Frequent loss of heterozygosity on chromosomes 1q, 5q, and 17p in human gastric carcinomas. *Cancer Res* **51**: 2926–2931

Schneider BG, Pulitzer DR, Brown RD, Prihoda TJ, Bostwick DG, Saldivar V, Rodrígez-Martínez HA, Gutiérrez-Díaz CME and O'Connell P (1995) Allelic imbalance in gastric cancer: an affected site on chromosome arm 3p. *Genes Chromosom Cancer* **13**: 263–271

Tahara E (1993) Molecular mechanism of stomach carcinogenesis. *J Cancer Res Clin Oncol* **119**: 1–8

Takahashi S, Qian J, Brown JA, Alcaraz A, Bostwick DG, Lieber MM and Jenkins RB (1994) Potential markers of prostate cancer aggressiveness detected by fluorescence *in situ* hybridization in needle biopsies. *Cancer Res* **54**: 3574–3579

Takahashi S, Shan AL, Ritland SR, Delacey KA, Bostwick DG, Lieber MM, Thibodeau SN and Jenkins RB (1995) Frequent loss of heterozygosity at

© Cancer Research Campaign 1997

7q31.1 in primary prostate cancer is associated with tumor aggressiveness and progression. *Cancer Res* **55**: 4114–4119

Tamura G, Kihana T, Nomura K, Terada M, Sugimura T and Hirohashi S (1991) Detection of frequent *p53* gene mutations in primary gastric cancer by cell sorting and polymerase chain reaction single-strand conformation polymorphism analysis. *Cancer Res* **51**: 3056–3058

Tamura G, Maesawa C, Suzuki Y, Tamada H, Satoh M, Ogasawara S, Kashiwaba M and Satodate R (1994) Mutations of the *APC* gene occur during early stages of gastric adenoma development. *Cancer Res* **54**: 1149–1151

Tamura G, Sakata K, Maesawa C, Suzuki Y, Terashima M, Satoh K, Sekiyama S, Suzuki A, Eda Y and Satodate R (1995) Microsatellite alterations in adenoma and differentiated adenocarcinoma of the stomach. *Cancer Res* **55**: 1933–1936

Tamura G, Ogasawara S, Nishizuka S, Sakata K, Maesawa C, Suzuki Y, Terashima M, Saito K and Satodate R (1996*a*) Two distinct regions of deletion on the long arm of chromosome 5 in differentiated adenocarcinomas of the stomach. *Cancer Res* **56**: 612–615

Tamura G, Sakata K, Nishizuka S, Maesawa C, Suzuki Y, Terashima M, Eda Y and Satodate R (1996*b*) Allelotype of adenoma and differentiated adenocarcinoma of the stomach. *J Pathol* **180**: 371–377

Tamura G, Sakata K, Nishizuka S, Maesawa C, Suzuki Y, Iwaya T, Terashima M, Saito K and Satodate R (1996*c*) Inactivation of the E-cadherin gene in primary gastric carcinomas and gastric carcinoma cell lines. *Jpn J Cancer Res* **87**: 1153–1159

Uchino S, Tsuda H, Noguchi M, Yokota J, Terada M, Saito T, Kobayashi M, Sugimura T and Hirohashi S (1992) Frequent loss of heterozygosity at the *DCC* locus in gastric cancer. *Cancer Res* **52**: 3099–3102

Visscher DW, Wallis TL and Crissman JD (1996) Evaluation of chromosome aneuploidy in tissue sections of preinvasive breast carcinomas using interphase cytogenetics. *Cancer* **77**: 315–320

Vogelstein B, Fearon ER, Hamilton SR, Kern SE, Preisinger AC, Leppert M, Nakamura Y, White R, Smits AMM and Bos JL (1988) Genetic alterations during colorectal-tumor development. *N Engl J Med* **319**: 525–532

Whelan SL, Parkin DM and Masuyer E eds (1993) *Trends in Cancer Incidence and Mortality*. IARC Scientific Publication No. 102. IARC: Lyon, France

Xiao S, Geng JS, Feng XL, Liu XQ, Liu QZ and Li P (1992) Cytogenetic studies of eight primary gastric cancers. *Cancer Genet Cytogenet* **58**: 79–84

Zenklusen JC, Oshimura M, Barrett JC and Conti CJ (1994*a*) Inhibition of tumorigenicity of a murine squamous cell carcinoma (SCC) cell line by a putative tumor suppressor gene on human chromosome 7. *Oncogene* **9**: 2817–2825

Zenklusen JC, Bièche I, Lidereau R and Conti CJ (1994*b*) $(C\text{-}A)_n$ microsatellite repeat *D7S522* is the most commonly deleted region in human primary breast cancer. *Proc Natl Acad Sci USA* **91**: 12155–12158

Zenklusen JC, Thompson JC, Troncoso P, Kagan J and Conti CJ (1994*c*) Loss of heterozygosity in human primary prostate carcinomas: a possible tumour suppressor gene at 7q31.1. *Cancer Res* **54**: 6370–6373

Zenklusen JC, Weitzel JN, Ball HG and Conti CJ (1995*a*) Allelic loss at 7q31.1 in human primary ovarian carcinomas suggests the existence of a tumor suppressor gene. *Oncogene* **11**: 359–363

Zenklusen JC, Thompson JC, Klein-Szanto AJP and Conti CJ (1995*b*) Frequent loss of heterozygosity in human primary squamous cell and colon carcinomas at 7q31.1: evidence for a broad range tumor suppressor gene. *Cancer Res* **55**: 1347–1350

© Cancer Research Campaign 1997